# Supplementary material for: Effect of relative weight limit set as a body weight percentage on work-related low back pain among workers
Source: PLoS One. 2023 Apr 19;18(4):e0284465. doi: 10.1371/journal.pone.0284465 (PMC10115297; doi:10.1371/journal.pone.0284465)
Supplement: Attachment — Submitted filename: Response_to_Reviewers.docx [file pone.0284465.s002.docx]

March 15, 2023

Dear Dr. Ng,

We thank the editor and referees for their careful reading of our manuscript and their helpful comments. We have revised our manuscript in response to the referees’ comments (Manuscript ID: PONE-D-22-34000).

We look forward to the publication of our manuscript in *PLOS ONE*.

Yours faithfully,

Kazuyuki Iwakiri

**--------------------------------------------------------------------------**

Our responses to the referees' reports are as follows. In addition to the points noted, we have changed the unclear sentences and erroneous descriptions. Moreover, we have added a dataset as a supporting information file and modified the figure resolution.

********************************************

**Response to Editor**

We greatly appreciate your consideration of our work. We are grateful for your comments.

**1. I particularly concur with comments from reviewer one. Please carry out the necessary improvement as per comments provided.**

**Response:** We have replied to every comment provided by reviewers.

**2. I am also intrigue with the methodology employed. Specifically, why was the load weight classified? What was the reference for the brackets of weight category?**

**Response:** We have changed the weight category to “no handling, 1–5 kg, 5–10 kg, 10–15 kg, 15–20 kg, 20–25 kg, 25–30 kg, and ≥30 kg.” The weight category was classified as every 5 kg since the weight limit of ISO is 25 kg.

**3. How did the analysis take into consideration of the severity of LBP?**

**Response:** In the multiple logistic regression, the dependent variable was severe LBP (non-severe and severe). The independent variable was body weight percentages (groups A, B, and C) or constant load weights (no handling, 1–5 kg, 5–10 kg, 10–15 kg, 15–20 kg, 20–25 kg, 25–30 kg, and ≥30 kg). The adjusted variables of the model were age, body height and weight, smoking status, industry, job demand and control, worksite social support, working posture, and postural change during work. We have added the relevant sentences in “Data analysis.” We did not perform multivariable logistic regression analysis with four grades of LBP as the dependent variable.

**[Changes in the revised manuscript]**

Lines 172–181:

The association between LBP (non-severe and severe) and body weight percentages (groups A, B, and C) or constant load weights (no handling, 1–5 kg, 5–10 kg, 10–15 kg, 15–20 kg, 20–25 kg, 25–30 kg, and ≥30 kg) was analyzed by multiple logistic regression using the forced entry method in which all parameters were forced into the model. Odds ratios (ORs) and 95% confidence intervals (95% CIs) were calculated for the model. The dependent variable was LBP, and the independent variable was body weight percentages or constant load weights. The adjusted variables of the model were age, body height and weight, smoking status, industry, job demand and control, worksite social support, working posture, and postural change during work. All variables were categorical data.

********************************************

**Response to reviewer 1**

We are grateful for your comments, which have helped to improve our paper.

**Reviewer #1:**

**Many valuable information was collected with an aim to understand the association between LBP and lifting risk variables. Although the authors examined the effectiveness of the current Japanese lifting standard using a percentage of body weight as the weight limit in reducing LBP, the authors did not explore other risk estimates using ISO or NIOSH methods. Most discussion does not add any value to the current science. What the study could have done is to use various risk calculation methods to determine which combination of the risk variables in the dataset is associated with the prevalence of LBP. The author could have even used some of the posture variables in combination of the weight limit as evidence for supporting the association. The suggested new findings may be used as the base for revising the Japan’s inadequate lifting standard using a percentage of body weight as the limit. The manuscript does have some merit that is worth publishing after a major revision that addresses the above general comment and the below specific comments.**

**Response:** We believe that ISO and NIOSH methods must be used in Japan since the weight limit based on body weight percentages (relative values) cannot suppress the prevalence of LBP. However, no studies have examined the effectiveness of relative values on LBP. We must prove that weight limits set as relative values are ineffective in preventing LBP. Therefore, this study investigated the effect of relative weight limits set as body weight percentages on LBP. After this study, we plan to examine whether the maximum weight and calculation formulas of ISO and NIOSH apply to Japanese people. We have added the relevant sentences in the Abstract, Introduction, and Discussion. Moreover, we have changed the title to “Effect of relative weight limit set as a body weight percentage on work-related low back pain among workers.”

**1. Line 24: the sentence “Moreover,……” in unclear. What are the categories of the constant load weights?**

**Response:** We have added the weight categories in the Abstract.

**[Changes in the revised manuscript]**

Lines 24–25:

Moreover, they were categorized into eight groups: no handling, 1–5 kg, 5–10 kg, 10–15 kg, 15–20 kg, 20–25 kg, 25–30 kg, and ≥30 kg.

**2. Line 111: Ten items are listed for examining job demands. If ten items were used, the lowest possible scores for job demands should be 10 assuming the Likert scale 1-4 was used. No variables are listed for job control and social support. Please add the info because these two additional variables were used in the study. What was the cut-off point of the score used for low and high stress? Please clarify.**

**Response:** Job demand questions are three items instead of ten items. Job control questions are three items, and worksite social support questions are six items. The score of the job demands, job control, and worksite social support was divided into low-stress and high-stress categories based on median values. Job demand and control scores ranged from 3 to 12; 3–7 points were defined as “low stress,” and 8–12 points were defined as “high stress.” Worksite social support scores ranged from 6 to 24; 6–15 points were defined as “low stress,” and 16–24 points were defined as “high stress.” We have changed the relevant sentences in “Job stressor.”

**[Changes in the revised manuscript]**

Lines 100–114:

Questions on job stressors were developed based on the three job demand items, three job control items, and six worksite social support items of the Brief Job Stress Questionnaire [21, 22]. Job demand parameters were assessed by “I have an extremely large amount of work to do,” “I can’t complete work in the required time,” and “I have to work as hard as I can.” Job control parameters were assessed by “I can work at my own pace,” “I can choose how and in what order to do my work,” and “I can reflect my opinion on workplace policy.” Worksite social support parameters were assessed by “How freely can you talk with the following people?” “How reliable are the following people when you are troubled?” and “How well will the following people listen to you when you ask for advice on personal matters?” The people were the respondent's superiors and coworkers. Responses were measured using the four-point scale. Total scores for job demand and control responses ranged from 3 to 12; 3–7 points were defined as “low stress,” and 8–12 points were defined as “high stress.” For the six worksite social support responses, scores ranged from 6 to 24; 6–15 points were defined as “low stress,” and 16–24 points were defined as “high stress.”

**3. Line 115: For clarity, I suggest the sentence from lines 92-93 for LBP be moved to LBP section (Lines 114-122).**

**Response:** The questions for which the time point is the first incidence of LBP include “working posture,” “manual handling status,” and “load weight handled regularly,” in addition to “LBP severity.” We left the sentence and added the time point of LBP in the LBP section.

**[Changes in the revised manuscript]**

Lines 116–118:

LBP was defined as pain in the lower back or buttocks lasting more than a day, including pain and numbness in the legs, which was the first incidence at the current job; however, this did not include back pain during menstruation, pregnancy or a cold.

**4. Line 124-128: Please specify if the posture and postural changes are for the trunk or torso, otherwise, the postures asked could mean hand, arm, and other body joints.**

**Response:** We have changed the relevant sentences in “Working posture and manual handling status.”

**[Changes in the revised manuscript]**

Lines 127–128:

Questions regarding working posture included working posture and postural change of the body trunk.

**5. Lines 155-158: Excluding the participants that performed push/pull or lifting more than 55 kgs is not justified well. Although there are recommended weight limits in various standard, participants who lifted more than 55 kg should be included in the analysis. Excluding pushing/pulling tasks is less concerning.**

**Response:** In the questionnaire, we asked participants to list the weight values per person handled by human power. As a result, 13453 workers were handling load weights of 1–56 kg, 713 were handling 56–100 kg, 629 were handling 100–500 kg, 57 were handling 500–1000 kg, 55 were handling 1000–5000 kg, and 15 were handling more than 5000 kg. Workers handling heavy weights may list the weight with a machine or the cumulative weight. To exclude these weights, this study used the 55kg weight limit of ILO. Pushing and pulling data were excluded as they will be analyzed separately from this study. We have added a description regarding load weight in “Weight of load.”

**[Changes in the revised manuscript]**

Lines 142–143:

Load weight was defined as the weight value per person handled by human power regularly.

**6. Line 167: Please explain what “forced entry metho” is? Add the reference groups for all the variables to the text, unless Tables have the info. Add a description for the dependent variables, such as linear or categorical multiple logistic regression. Technically the constant load weight could be modeled as linear continuous variables for their linear intervals.**

**Response:** We have changed “forced entry metho” to “the forced entry method in which all parameters were forced into the model.” We have added “reference” to an OR of 1.00 in Tables 3 and 4. Moreover, we added descriptions regarding the dependent, independent, and adjusted variables in “Data analysis.” We analyzed constant load weights as a continuous variable. The OR of LBP for male workers was 1.02 (1.01–1.02), and for female workers was 1.03 (1.02–1.05). The results indicate that an increase of 1 kg increases LBP risk by 1.02 for male workers and 1.03 for female workers. However, since this is difficult to understand, we used eight weight categories.

**[Changes in the revised manuscript]**

Lines 177–181:

The dependent variable was LBP, and the independent variable was body weight percentages or constant load weights. The adjusted variables of the model were age, body height and weight, smoking status, industry, job demand and control, worksite social support, working posture, and postural change during work. All variables were categorical data.

**7. Line 178-183: Text may be removed because all the presented info is in Figure 1. Suggest that the authors simple describe the total sample size that met the inclusion criteria.**

**Response:** We have changed the relevant sentence in “Target of analysis.”

**[Changes in the revised manuscript]**

Lines 187–189:

Completed questionnaires were collected from 21924 workers, including 14779 males and 7145 females (Fig 1). Of them, the male workers of groups A to C were 9607, 3623, and 1549, and the female workers were 5428, 1019, and 698, respectively.

**8. Table 1: Change “body height” to statue. Ad a footnote to define part-time. Recommend that the authors add % or mean to each variable because they are not very obvious.** **The scores of the job demands, control and social support have little clinical meaning. Suggest that the author change them to low and high using the medium cut-off point or Japanese stress standard in the literature for classifying the stress level. Then percentage of low or high level for each variable can be presented. Chi-square test can be further used for comparing pairs of the different levels of each variables. For example, the job control in female Group A may be different from Group B and C, but there is no difference between Groups B and C.**

**Response:** Since our research fields of occupational health and ergonomics use “body height” instead of “statue,” we want to use “body height.” We have added a footnote, “Part-timer and temporary worker,” to define part-time in Table 1. Moreover, we have changed the notation of “mean ± SD” and “%” in Tables 1 and 2.

The score of the job demands, job control, and worksite social support was divided into low-stress and high-stress categories based on median values and was reanalyzed by multiple logistic regression. We added the relevant sentence in “Job stressor” and “Basic information of workers” and presented the results in Tables 1, 3, and 4.

**[Changes in the revised manuscript]**

Lines 110–114:

Total scores for job demand and control responses ranged from 3 to 12; 3–7 points were defined as “low stress,” and 8–12 points were defined as “high stress.” For the six worksite social support responses, scores ranged from 6 to 24; 6–15 points were defined as “low stress,” and 16–24 points were defined as “high stress.”

Lines 204–207:

For both sexes, occupational stress regarding job demand was greater in group B than in group A and greater in group C than in group B. Job control was greater in groups B and C than in group A. Worksite social support for male workers was greater in group B than in groups A and C.

**9. Line 209: Improper posture is not an academic term. Suggest that the authors use non-neutral posture if this is what it meant in the questionnaire. A larger number sounds better than a higher number. Suggest that the authors change the expression throughout the manuscript. Chang the section title to “Work** **characteristics”.**

**Response:** We have changed “improper posture” to “non-neutral posture,” “proper posture” to “neutral posture,” “higher” to “greater,” and “Work description” to “Work characteristics” in all text and tables. Moreover, we have added a description of the non-neutral and neutral postures in “Working posture and manual handling status.”

**[Changes in the revised manuscript]**

Lines 131–133:

The neutral posture was defined as a straight-backed posture without excessive force, whereas the non-neutral posture was described as other unnatural positions.

**10. Line 228: Because the first episode of the LBP was recorded in a retrospective manner regardless of the length of recall time (i.e., could be one month or 10 years). It may be inappropriate to describe the reporting of LBP as simply prevalence. Consider using a revised term career-time cumulative prevalence of LBP.**

**Response:** We agree with the reviewer’s comment but think “career-time cumulative prevalence” is too long. We have added a description of severe LBP prevalence in “Low back pain.”

**[Changes in the revised manuscript]**

Lines 123–124:

The prevalence of severe LBP was calculated as a percentage of LBP caused by career-time cumulative burden.

**11. Table 3 title is misleading because only the bottom three lines have the body weight percentage limit information. The reference group for each variable is different and nothing to do with the body weight limit. Table 3 needs to be revised to show proper comparisons. Similarly, Table 4 needs to be revised according to the above question.**

**Response:** Per the reviewer's comment, we have changed Tables 3 and 4. The independent variables are listed at the top of the tables, and the adjusted variables are listed below labeled “adjusted variables.”

**12. Line 266: The statement “For percentages of body weight, the prevalence of severe LBP was lower in group B than in group C but higher than in group A. These associations were similar for the adjusted ORs of LBP.” May be the most important finding of the study. However, no discussion is provided for why the moderate load in term of body weight limit had the highest prevalence of LBP. The second paragraph in Discussion provides little interpretation of the results. It reads like results. The third paragraph discusses BMI, but the preceding paragraph (2nd) does not have BMI info. BMI data do support the argument. The higher BMI in Group B should be used for discussion.**

**Response:** LBP prevalence in group B was not the greatest and was smaller than in group C. However, the prevalence was greater than those of group A. These results indicate that the weight limit in group B had some preventive effects on LBP, but the preventive effects on LBP caused by handling loads were insufficient. Moreover, from a biomechanical point of view, limiting weights as body weight percentages are problematic. On the other hand, limiting weight under 10 kg suppressed the development of LBP caused by handling loads. Therefore, relative weight limits set as body weight percentages were inappropriate and ineffective for preventing LBP. We have changed the relevant sentence in the Discussion.

In this study, greater BMI had a high prevalence of LBP; 33.6% of male workers with ≥25.0 BMI, 30.7% with 18.5–25.0 BMI, and 21.8% with <18.5 BMI had LBP. Similarly, 25.8% of female workers with ≥25.0 BMI, 20.1% with 8.5–25.0 BMI, and 17.9% with <18.5 BMI had LBP. However, high and low BMI groups are not the same height and cannot be compared. We want to omit the description of BMI.

**[Changes in the revised manuscript]**

Lines 281–294:

Limiting weight to 40% for male workers and 24% for female workers or less of body weight had some preventive effects on LBP. However, the preventive effects on LBP caused by handling loads were insufficient since their LBP risk was greater than those of no handling loads. From a biomechanical point of view, limiting weights as body weight percentages are problematic. When people of the same height and different weights take the same posture, the shearing force on the lumbar disc is greater in a heavier than in a lighter person [18, 19]. This is because the extra weight in a heavier person increases the shearing force on the lumbar disc. In addition, with the weight limit set as body weight percentages, a heavier person can lift heavier loads, increasing the shear force on the lumbar disc. Conversely, limiting weight under 10 kg suppressed the development of LBP caused by handling loads. Therefore, relative weight limits set as body weight percentages were inappropriate and ineffective for preventing LBP. Workers’ LBP may be suppressed by an absolute load weight rather than a relative load weight.

**13. Line 288: The sentence “This study showed that handling loads of 10 kg or more could not suppress severe LBP.” does not make sense. The following discussion shows mixed effect of lifted weight on the development of LBP. Both ISO and NIOSH use lifted load and postural information to determine the risk of LBP. It’s a scientific consensus that both are important lifting risk information. Using load alone is not a good risk estimate.**

**Response:** We have changed the relevant sentence in the Discussion. We believe that only limiting the lifting weight cannot prevent LBP. In particular, working posture is an important factor related to LBP. Hence, in this study, working posture was added to the adjusted variable and analyzed. As a result, this study showed that a weight of 10 kg might be a threshold, even considering working postures. This paragraph discusses whether a weight of 10 kg is a threshold.

**[Changes in the revised manuscript]**

Lines 295–296:

In this study, a load weight of 10 kg was the threshold for LBP caused by handling loads.

********************************************

**Response to reviewer 2**

Thank you for your valuable comments and advice.

**1. Abstract:**

**Gender representation is mixed in terms of Men/women and Male/Female. It would be better to unify them.**

**Response:** We have changed “men” to “male workers” and “women” to “female workers” in all text.

**2. Basic information of workers**

**Duration of occupational engagement may influence the onset of low back pain. The authors should add to Basic information if possible.**

**Response:** We agree with the reviewer’s comment. However, we have not collected the duration of occupational engagement.

**3. Work description**

**Line 220-221：「In the three groups, 82.7% of male and 86.0% of female workers had no or a small effect due to COVID-19 on their work」Data showing this statement is not included in the table 2, it would be better to add it.**

**Response:** We have added the impact of COVID-19 in Table 2 and changed the relevant sentences in "Work characteristics."

**[Changes in the revised manuscript]**

Lines 228–229:

Approximately 80% of male and female workers had no or a small effect on their work due to COVID-19.
